# Syphilis by Vaccination with Human Virus

**Published:** 1879-10

**Authors:** 


SYPHILIS BY VACCINATION WITH HUMAN VIRUS.

VIRUS was taken from the seven months' old child of a mother, subsequently known to be syphilitic, the infant at the time presenting all the appearances of perfect health.

With the virus thus obtained, twenty-five girls were vaccinated, twelve of whom in six weeks had ulcerations at the point of inoculation, succeeded by exanthem, oral and pharyngeal ulcers, and condylomata, syphilitic ozœna, etc. Three others had suspicious lesions in the neighborhood of the vaccine sore, which were not followed by constitutional symptoms.—*Gazette des Hospitaux.*

